# Racial Disparities in Mortality Associated with Systemic Lupus Erythematosus — Fulton and DeKalb Counties, Georgia, 2002–2016

**DOI:** 10.15585/mmwr.mm6818a4

**Published:** 2019-05-10

**Authors:** S. Sam Lim, Charles G. Helmick, Gaobin Bao, Jennifer Hootman, Rana Bayakly, Caroline Gordon, Cristina Drenkard

**Affiliations:** ^1^Division of Rheumatology, Department of Medicine, Emory University, Atlanta, Georgia; ^2^Division of Population Health, National Center for Chronic Disease Prevention and Health Promotion, CDC; ^3^Georgia Department of Public Health; ^4^Rheumatology Research Group, Institute of Inflammation and Ageing, University of Birmingham, United Kingdom.

Systemic lupus erythematosus (SLE) is a chronic, systemic autoimmune disease with often nonspecific symptoms that can lead to a delay in diagnosis. The disease disproportionately affects women and minorities. Blacks with SLE also have more severe disease and develop it at an earlier age (*1*). Despite an increase in the 5-year survival rate from 50% in 1955 to approximately 90% in the 2000s, attributed largely to advances in management of SLE (*2*), premature mortality among SLE patients persists, often as a result of disease severity, infections, and cardiovascular disease. Because existing SLE mortality estimates based on death certificate data are known to underestimate SLE deaths (*3*), SLE mortality was analyzed using 2002–2004 data from the population-based Georgia Lupus Registry (*1*). Incident and prevalent SLE cases matched to the National Death Index through 2016 identified 97 and 401 deaths, respectively. Standardized mortality ratios adjusted for age group, sex, and race were two to three times higher among persons with SLE relative to expected deaths in the general population. Blacks had significantly higher cumulative mortality than did whites, and blacks with both incident and prevalent cases were significantly younger at death (mean age 51.8 and 52.3 years, respectively) than were whites (mean age 64.4 and 65.0 years, respectively). Whites had lower mortality after diagnosis than did blacks; among incident cases, mortality among whites did not occur until 5 years after SLE diagnosis, whereas blacks had significantly and persistently higher mortality from the time of diagnosis. There were no significant differences by sex. Current CDC-supported efforts encourage early detection, diagnosis, and treatment, and enhanced self-management skills to mitigate racial disparities and improve outcomes overall among persons with SLE.

The Georgia Lupus Registry (*1*) was designed to collect data on all residents of two Georgia counties (Fulton and DeKalb) in the Atlanta metropolitan area with large black and white populations. The public health surveillance exemption to the Health Insurance Portability and Accountability Act Privacy Rule (https://www.hhs.gov/hipaa/for-professionals/privacy/index.html) allowed investigators to obtain protected health information (PHI) without written consent of the patient. Application of this exemption enabled investigators to ascertain all potential cases, determine whether potential cases met case definition criteria, and provide enough information to prevent duplicate counting of patients examined in multiple facilities. PHI was stored securely, and its use was limited to authorized research personnel, maximizing the use of deidentified data whenever feasible.

The primary sources of potential cases included hospitals, rheumatologists, nephrology groups, and dermatology groups in and around the two counties. Administrative databases were queried retrospectively for billing codes for lupus and related conditions. Secondary sources included laboratories (including pathology laboratories) and queries in other population databases (*1*). Abstractors were trained and underwent regular quality assessments. The study was reviewed and approved by the Institutional Review Boards at Emory University and the Georgia Department of Public Health. CDC determined this study did not meet the definition of human subjects research (public health practice). SLE prevalence was estimated for 2002 and incidence for 2002–2004 from the Georgia Lupus Registry. Denominator data for the two counties were obtained from postcensal population estimates. Age-adjusted estimates and 95% confidence intervals were calculated based on the standard 2000 projected age distribution (*1*).

A case of SLE was defined as meeting either the 1997 update of the 1982 American College of Rheumatology (ACR) revised classification criteria (meeting four or more of the 11 criteria*) (*4*,*5*) or an alternative definition (three of the ACR criteria plus a documented diagnosis of SLE by the patient’s board-certified rheumatologist). All incident and prevalent SLE cases were matched to the National Death Index through 2016. Cause of death codes were available but not analyzed because of poor reliability regarding SLE attribution (*3*). Standardized mortality ratios were calculated as the ratio of observed deaths among persons with prevalent SLE to expected deaths in the general county populations; subgroups were compared using the same age group, sex, and race categories. The number of expected deaths was calculated by multiplying the death rate of the general population in Fulton and DeKalb counties by the total number of SLE patients in each group. There were too few deaths to calculate standardized mortality ratios for the incident SLE group. Cumulative mortality used Kaplan-Meier survival analysis for both incident and prevalent cases to determine the percentage of SLE patients dying since their diagnosis (*1*). Analyses were performed using SAS software (version 9.4; SAS Institute).

During 2002–2004, a total of 336 incident SLE cases were identified; these SLE patients were demographically similar to the patients in 1,353 cases with prevalent SLE in 2002 (87%–90% female, 74%–76% black, and 23% white) but were older at SLE diagnosis (mean age 40.6 years) than were patients with prevalent SLE (34.6 years). Among patients with prevalent and incident SLE, 401 and 97 deaths, respectively, occurred through 2016. Standardized mortality ratios using 2002–2016 data were 2.3–3.3 times higher for persons with prevalent SLE relative to expected deaths in the general population (Table). Black females with prevalent SLE were three times more likely to die than were black females in the general population (standardized mortality ratio = 3.38). Cumulative mortality was significantly higher among blacks than among whites for both incident (Figure 1) and prevalent (Figure 2) SLE; death occurred at a younger age among blacks with incident SLE cases (mean age = 51.8 ± 17.5 years) and prevalent SLE cases (mean 52.3 ± 15.9 years) than it did among whites (64.4 ± 18.9 years and 65.0 ± 16.3 years, respectively) (p<0.001). Mortality among whites was markedly lower in the years immediately following diagnosis compared with mortality among blacks; among incident cases, no deaths were observed among whites until 5 years after SLE diagnosis, whereas mortality among blacks was persistently higher from the time of diagnosis. In addition, whites with SLE had the same cumulative mortality proportion (9%) in 10 years as that observed in blacks in 2 years (Figure 1). There were no significant differences by sex.

**TABLE:** Standardized mortality ratios for patients with prevalent cases of systemic lupus erythematosus (SLE) from 2002 to 2016, adjusted by age, sex, and black/white race* — Georgia Lupus Registry

Characteristic	No. of SLE patients (%)	Deaths		Standardized mortality ratio (95% CI)
		Observed	Expected	
**Overall (black and white^†^)**	**1,335 (100)**	**400**	**128**	**3.12 (2.83–3.44)**
**Sex**				
Male	135 (10.1)	51	17	2.98 (2.27–3.92)
Female	1,200 (89.9)	349	111	3.14 (2.83–3.49)
**Race**				
Black	1,024 (76.7)	324	97	3.34 (3.00–3.72)
White	311 (23.3)	76	31	2.43 (1.94–3.04)
**Race/Sex (total = 1,200)**				
Black female	924 (77.0)	287	85	3.38 (3.01–3.79)
White female	276 (23.0)	62	26	2.36 (1.84–3.02)

**Abbreviation:** CI = confidence interval.

* Age on July 1 was used for adjustment. The standardized mortality ratio is a ratio between the observed number of deaths in those with SLE and the number of deaths expected, based on age, sex, and race specific rates in Fulton and DeKalb counties. CIs are based on a generalized estimating equation model with Poisson distribution.

^†^ Eighteen persons who were not identified as black or white, including one who died, were excluded.

**FIGURE 1:**
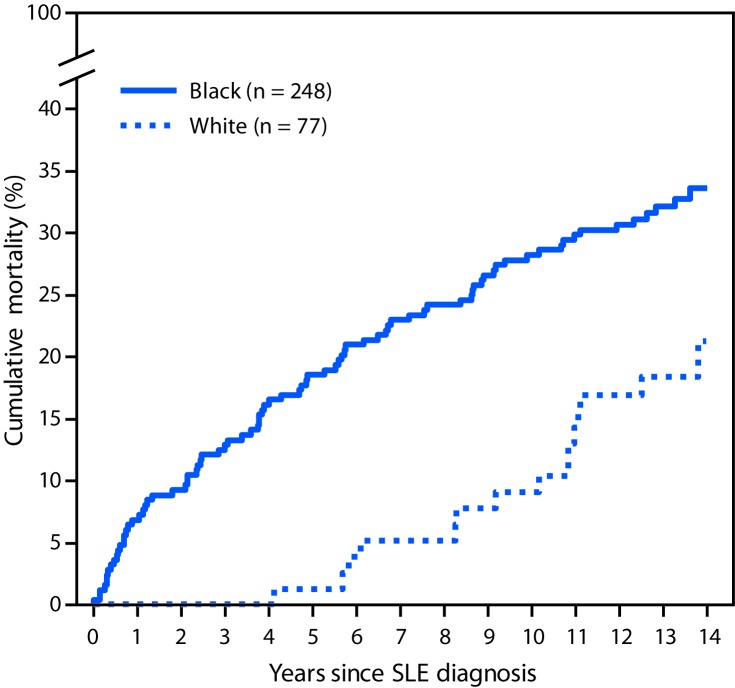
Cumulative mortality* of incident systemic lupus erythematosus (SLE) cases diagnosed during 2002–2004, by black/white race — Georgia Lupus Registry, 2002–2016 * Cumulative mortality for incident SLE cases was calculated using Kaplan-Meier survival analysis to indicate the probability of SLE patients dying at a specified time since diagnosis. Difference p = 0.008, by log rank test.

**FIGURE 2:**
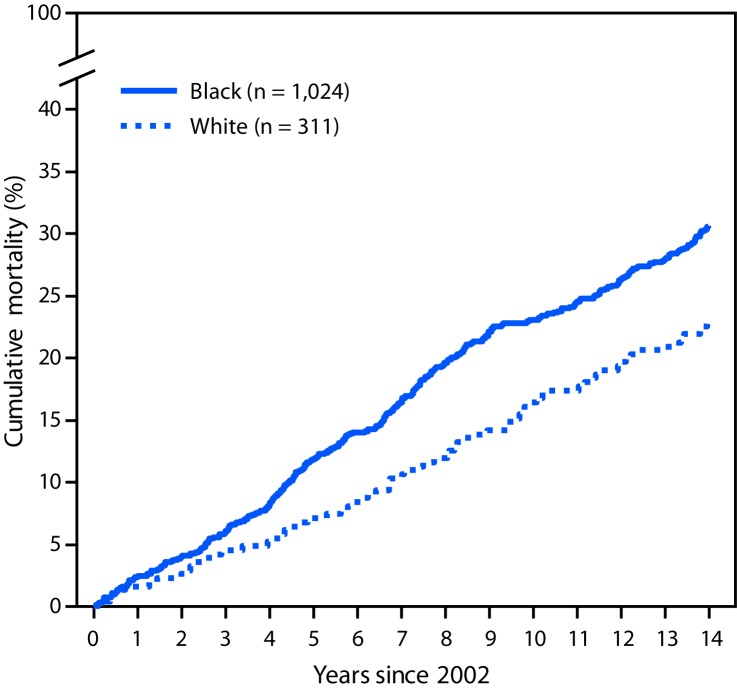
Cumulative mortality* of prevalent systemic lupus erythematosus (SLE) cases diagnosed in 2002, by black/white race — Georgia Lupus Registry, 2002–2016 * Cumulative mortality for prevalent SLE cases was calculated using Kaplan-Meier survival analysis to indicate the probability of SLE patients dying at a specified time since 2002. Difference p = 0.025, by log rank test.

## Discussion

Despite increasing awareness of SLE and advancements in treatment (*6*), mortality among persons with SLE remains high, with the highest standardized mortality ratio among black females. The effect of this racial disparity in mortality is further underscored by the fact that the prevalence of SLE in blacks is three times that in whites (*1*).

These findings are similar to those reported in a 2002 study, which also found a higher incidence and prevalence among women and blacks, but the current study used more accurate methods to ascertain cases (*7*). A recent nationwide study using causes of death from 1968 through 2013 obtained from death certificate data in CDC’s WONDER database (https://wonder.cdc.gov) showed that age-standardized mortality rates decreased over time among SLE patients but remained high relative to non-SLE mortality, with the highest mortality rates in women, blacks, and residents of the South and West U.S. Census regions (*8*). Both of these studies depended solely on death certificates to identify cases of SLE, which only capture an estimated 40%–60% of SLE cases (*3*,*9*).

The findings in this report are subject to at least four limitations. First, racial identity was assigned based primarily on the physician’s assessment as documented in the medical record, which might not reflect the patient’s self-identity. Second, some cases might have been missed in the original registry. Third, there might be variability in SLE diagnosis by rheumatologists, and undiagnosed cases were not sought. Finally, these results might not be generalizable outside the two counties. Strengths of the current study include the use of a population-based lupus registry identifying nearly all validated SLE cases in the two-county area and the long follow-up period, resulting in data on more SLE deaths than would be identified by death certificate diagnoses alone.

Prioritizing the identification of reversible mortality factors and developing strategies to address them could aid in mitigating racial disparities and improving outcomes overall in SLE. The first-ever National Public Health Agenda for Lupus (*10*) describes a plan to address lupus from a public health perspective. Other CDC-supported, population-based lupus registries and longitudinal follow-up activities include examining natural history, treatment, access to care, and disparities as potential factors in SLE mortality and progression (https://www.cdc.gov/lupus/funded/lupus-studies.htm). The Lupus Foundation of America and the American College of Rheumatology are working together to encourage early detection and treatment of lupus, enhance the self-management skills of patients with lupus, and improve health care providers’ ability to make accurate diagnoses. Additional information is available at https://www.cdc.gov/lupus/funded/awareness.htm.

**Summary**

**What is already known about this topic?**

Systemic lupus erythematosus (SLE) is a systemic autoimmune disease that disproportionately affects women and minorities. The 5-year survival rate of patients with SLE has been improving.

**What is added by this report?**

Using improved methods by following SLE patients carefully defined in a population-based registry, standardized mortality ratios were two to three times higher in persons with SLE than in the general population. Compared with whites with SLE, cumulative SLE mortality was significantly higher among blacks, with deaths occurring sooner after diagnosis and at a mean age approximately 13 years younger.

**What are the implications for public health practice?**

Current CDC-supported efforts to encourage early detection, diagnosis, and treatment, and to enhance self-management skills might mitigate racial disparities and improve overall outcomes in SLE.

## Footnotes

* The 11 criteria are as follows: malar rash, discoid rash, photosensitivity, oral ulcers, arthritis, serositis (pericarditis or pleuritis), renal disorder, neurologic disorder, hematologic disorder, immunologic disorder, and antinuclear antibody. https://www.rheumatology.org/Portals/0/Files/1997%20Update%20of%201982%20Revised.pdf.
